# Halogenoderma: A Case Report and Review of the Literature

**DOI:** 10.7759/cureus.31846

**Published:** 2022-11-23

**Authors:** Raghad A Ghazzawi, Mohammed G Alqurashi, Nada A Almalki, Ashwaq K Alosaimi, Khalid Al Hawsawi

**Affiliations:** 1 Medicine and Surgery, King Faisal Specialist Hospital and Research Centre, Jeddah, SAU; 2 Internal Medicine, King Saud Bin Abdulaziz University for Health Sciences College of Medicine, Jeddah, SAU; 3 Medicine and Surgery, Taif University, Taif, SAU; 4 Dermatology, Umm Al-Qura University, Makkah, SAU; 5 Dermatology, King Abdulaziz Hospital, Makkah, SAU

**Keywords:** bromide, iodide, bromoderma, iododerma, halogenoderma

## Abstract

Halogenoderma (HD) is an uncommon dermatosis that develops following exposure to halogens such as iodide and bromide, referred to as iododerma and bromoderma, respectively. Here, we report the case of a 40-year-old male who presented with a three-week history of slightly itchy progressive skin lesions associated with low-grade fever and malaise. The patient had a history of using food supplements containing iodide and bromide for four months prior to the appearance of skin rashes. Skin examination revealed multiple crusted papules and nodules scattered on his face, neck, and trunk. A skin biopsy was taken from the lesions. The epidermis showed crustation, exocytosis of neutrophils, and multiple intraepidermal abscesses. The dermis showed heavy cellular infiltrates composed mainly of neutrophils. The skin lesions disappeared completely after the cessation of food supplements, along with the use of topical corticosteroids for a few weeks.

## Introduction

Halogenoderma (HD) is an uncommon dermatosis that develops following exposure to halogens such as iodide and bromide, referred to as iododerma and bromoderma, respectively. Patients with HD present mainly with pustules or papulopustular lesions frequently located on the face, neck, back, and extremities [1]. HD can sometimes present as extensive vegetating lesions rather than pustular eruptions [2].

The exact pathogenesis of HD remains unknown. It is believed to be caused by a type 2 delayed hypersensitivity reaction [3]. The treatment of HD includes the avoidance of intake of iodide- or bromide-containing substances, and lesions usually spontaneously disappear after four to six weeks following the stopping of iodide or bromide intake [3]. Systemic corticosteroids can be used for more rapid healing of these lesions [4].

Here, we present a rare case of HD in a 40-year-old male who presented with multiple crusted papules and nodules scattered on his face, neck, and trunk following the use of food supplements containing iodide and bromide.

## Case presentation

**Figure 1:**
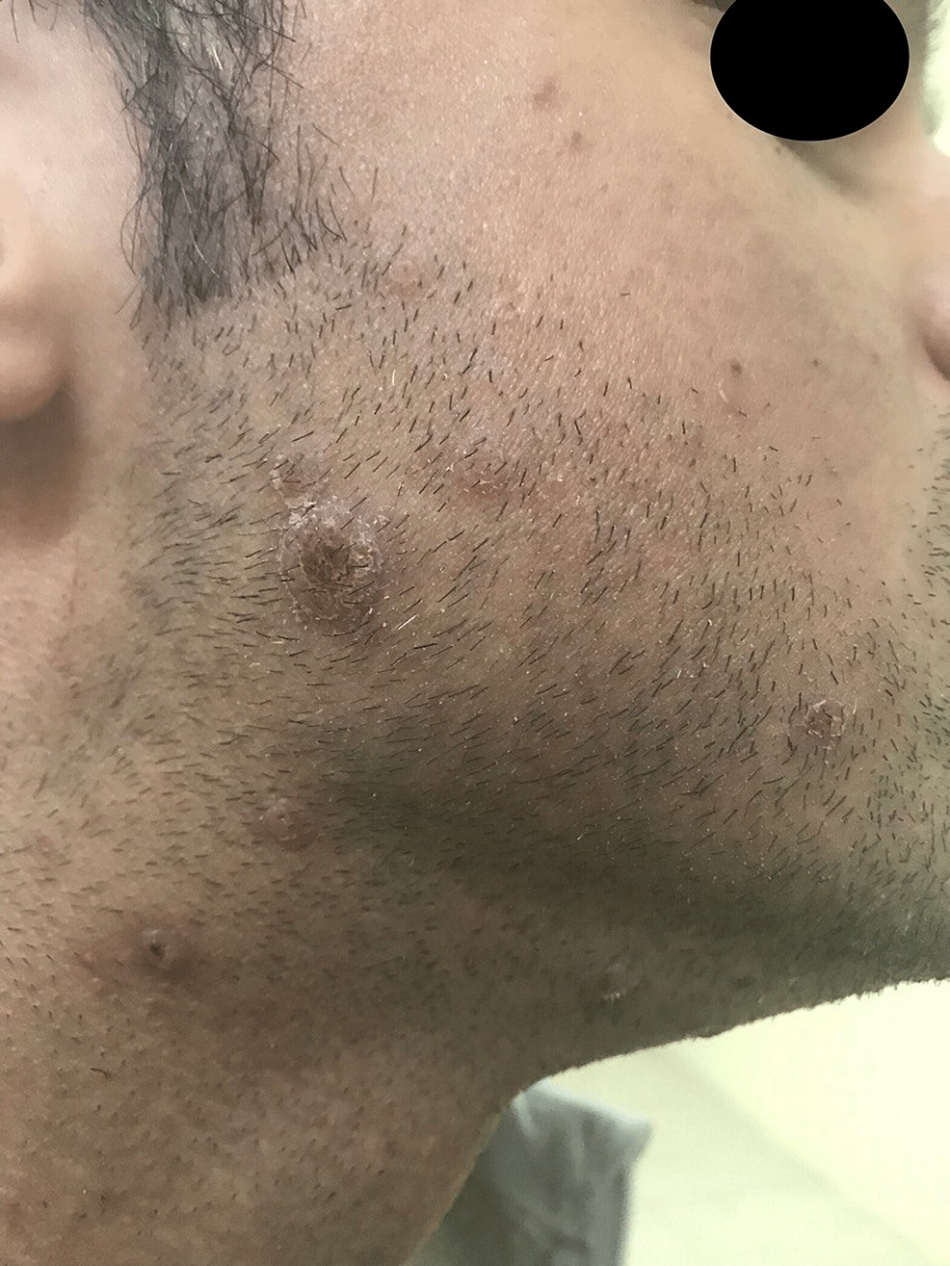
Multiple crusted papules and nodules scattered on the patient’s face and neck.

A 40-year-old male presented with a three-week history of slightly itchy progressive skin lesions associated with fever and malaise at the beginning and then disappearing. There were no similar attacks before and no history of contact with a similar condition. The patient had a positive history of using iodine and bromide food supplements for four months prior to the appearance of skin rashes. An allergy history, surgical history, medical history, family history, and review of systems were all unremarkable. The patient did not experience any symptoms of iodism such as mouth burning or increased salivation, metallic taste, tooth and gum soreness, and headache. Skin examination revealed multiple crusted papules and nodules scattered on his face, neck, and trunk (Figure 1).

**Figure 2:**
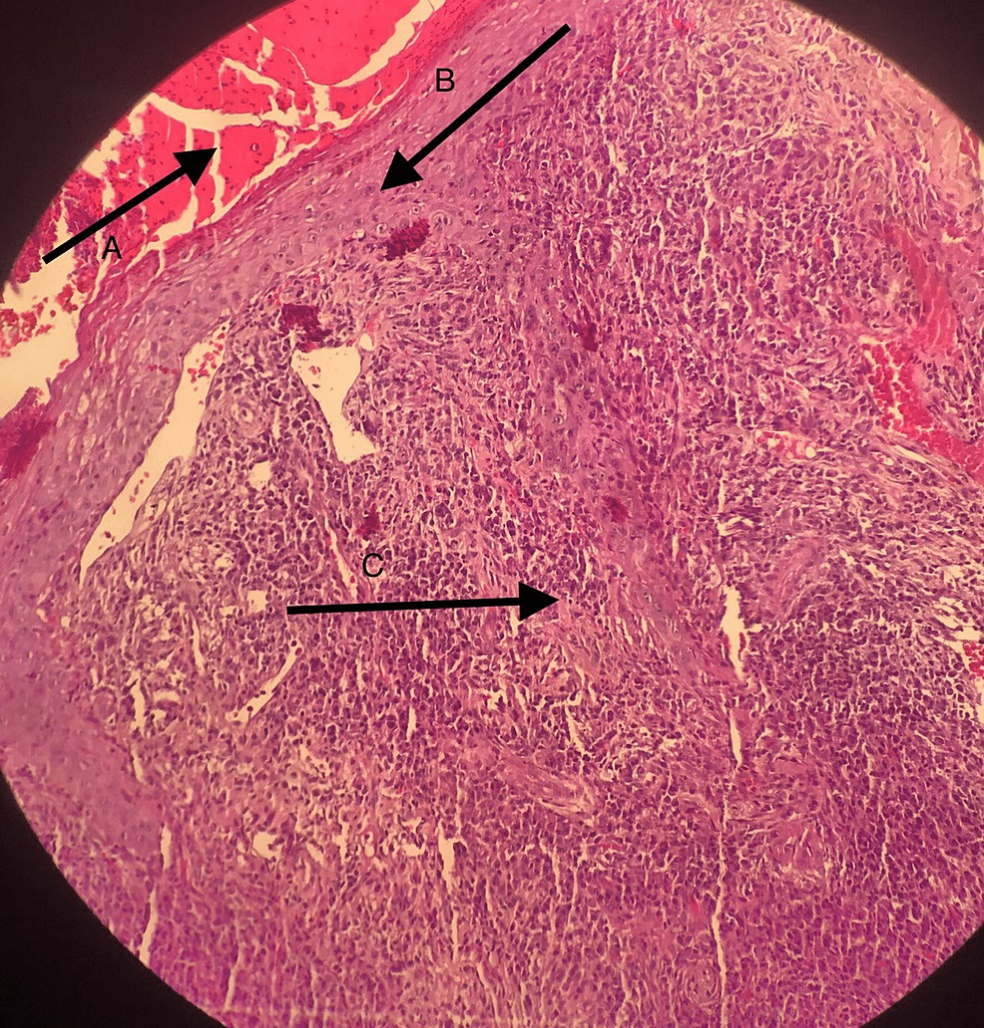
A skin biopsy showing epidermal crustation (A) and exocytosis of neutrophils (B). The dermis showed heavy cellular infiltrates composed mainly of neutrophils (C) (hematoxylin and eosin stain; original magnification, ×20).

Hair, nail, and mucus membrane examinations were all normal. Differential diagnoses included chicken pox, pityriasis lichenoides varioliformis acuta, dermatitis herpetiformis, Sweet syndrome, bacterial folliculitis, and lymphomatoid papulosis. A skin biopsy of the lesion was performed. The epidermis showed crustation, exocytosis of neutrophils, and multiple intraepidermal abscesses. The dermis showed heavy cellular infiltrates composed mainly of neutrophils (Figure 2).

Based on the above clinicopathological findings, the patient was diagnosed with HD. The patient was reassured and told to stop using the food supplements. A topical corticosteroid was prescribed. One month after using the topical corticosteroid, the lesions disappeared with no recurrence at the time of this report.

## Discussion

HD is an uncommon inflammatory dermatosis that develops following exposure to halogens such as iodide and bromide. Iodide is found in seaweed, salt, amiodarone, radiocontrast media, and potassium iodide, and rarely as topical iodide use [3,5]. Iododerma usually appears following the systemic use of potassium iodides, such as in the case of asthma, Graves’ disease, and bronchitis [1]. Bromide is found in some drugs such as bromocriptine, analgesics, and hypnotics [6]. Bromide is also found in topical preparations, such as methyl bromide, which is used by farmers for feeding animals [7]. Both iodide and bromide are found in food supplements [5]. Iododerma commonly presents as acneiform lesions and rarely as vesicular, pustular, hemorrhagic, urticarial, fungating, suppurative, nodular, or ulcerative lesions on the face [3], whereas bromoderma commonly presents as verrucous, ulcerating plaques on the lower extremities [5,7].

Aliagaoglu et al. reported a case of iododerma following the topical use of a povidone-iodine solution for disinfection purposes at work [4]. Another study reported iododerma in a pregnant female who was taking iodine-containing multivitamins to decrease the risk of neural tube defects during pregnancy [8]. Young and Grossman reported a case of acute iododerma after receiving 1,942 g of iodine within three days after undergoing contrast CT [9]. In our study, the patient had been using oral food supplements with iodine and bromide for muscle-building purposes for four months prior to the onset of his skin eruptions. HD can mimic other skin conditions that have similarities in both clinical and histological findings. These include Sweet syndrome and pyoderma gangrenosum [10,11]. However, our patient showed the classical histopathological features of HD. Our patient did not have any manifestations of iodism such as increased salivation, metallic taste, tooth and gum soreness, and headache.

The main treatment of HD is the complete cessation of the causative agent and the use of topical or systemic corticosteroids [2,3]. A dramatic response has been observed after using a systemic corticosteroid in a patient with extensive HD [12]. Silver sulfadiazine cream (a topical antibiotic) with topical corticosteroids has been used successfully in a patient with extensive vegetative bromoderma [2]. Our patient stopped using the food supplements and responded well after four weeks of topical corticosteroid use. At follow-up, there was no recurrence at the time of writing this report.

## Conclusions

HD is a rare skin eruption that develops following exposure to halogens such as iodide and bromide, referred to as iododerma and bromoderma, respectively. HD should be considered in patients with a history of iodine and bromide use presenting with papulopustular lesions, and it should be carefully distinguished from other skin conditions that mimic the clinical and histological appearance of HD.
